# Implications of Projected Hydroclimatic Change for Tularemia Outbreaks in High-Risk Areas across Sweden

**DOI:** 10.3390/ijerph17186786

**Published:** 2020-09-17

**Authors:** Yan Ma, Guillaume Vigouroux, Zahra Kalantari, Romain Goldenberg, Georgia Destouni

**Affiliations:** 1 Department of Physical Geography, Stockholm University, 106 91 Stockholm, Sweden; guillaume.vigouroux@natgeo.su.se (G.V.); zahra.kalantari@natgeo.su.se (Z.K.); romain.goldenberg@natgeo.su.se (R.G.); georgia.destouni@natgeo.su.se (G.D.); 2 Bolin Centre for Climate Research, Stockholm University, 106 91 Stockholm, Sweden

**Keywords:** hydroclimatic change, infectious disease, tularemia, CMIP6 projections, high-risk sites, Sweden

## Abstract

Hydroclimatic change may affect the range of some infectious diseases, including tularemia. Previous studies have investigated associations between tularemia incidence and climate variables, with some also establishing quantitative statistical disease models based on historical data, but studies considering future climate projections are scarce. This study has used and combined hydro-climatic projection outputs from multiple global climate models (GCMs) in phase six of the Coupled Model Intercomparison Project (CMIP6), and site-specific, parameterized statistical tularemia models, which all imply some type of power-law scaling with preceding-year tularemia cases, to assess possible future trends in disease outbreaks for six counties across Sweden, known to include tularemia high-risk areas. Three radiative forcing (emissions) scenarios are considered for climate change projection until year 2100, incuding low (2.6 Wm^−2^), medium (4.5 Wm^−2^), and high (8.5 Wm^−2^) forcing. The results show highly divergent changes in future disease outbreaks among Swedish counties, depending primarily on site-specific type of the best-fit disease power-law scaling characteristics of (mostly positive, in one case negative) sub- or super-linearity. Results also show that scenarios of steeper future climate warming do not necessarily lead to steeper increase of future disease outbreaks. Along a latitudinal gradient, the likely most realistic medium climate forcing scenario indicates future disease decreases (intermittent or overall) for the relatively southern Swedish counties Örebro and Gävleborg (Ockelbo), respectively, and disease increases of considerable or high degree for the intermediate (Dalarna, Gävleborg (Ljusdal)) and more northern (Jämtland, Norrbotten; along with the more southern Värmland exception) counties, respectively.

## 1. Introduction

Climate change may cause shifts in geographical range, prevalence, and/or severity of some infectious diseases [1,2,3,4,5], including tularemia [6,7], a dangerous zoonotic disease caused by the intracellular bacterium *Francisella tularensis* [8] and widely prevalent in Europe, Asia, and America [8]. Transmission of Tularemia is usually caused by contact with infected rodents and hares, or by arthropod vectors [9]. In Europe, there is also a strong association between *F. tularensis subsp. holarctica* (Type B) and water conditions, with many humans reported to have contracted the disease around lakes and rivers [10]. Europe as a whole does not have a clear trend of tularemia outbreaks in recent decades, but rather a pattern of repeated local emergence and re-emergence throughout most countries [11]. In Sweden, however, the nationwide incidence of tularemia increased during the period 1984-2012 and the disease now occurs over a larger geographical area [12].

Previous studies have reported relationships between hydroclimatic factors and tularemia outbreaks [10,13,14,15,16,17], with some also evaluating future change scenarios [10,15,16]. However, perspectives and conclusions regarding future tularemia changes vary. For example, for tularemia in Sweden, Palo et al. [16] concluded that warming should not increase the frequency of tularemia outbreaks, whereas Rydén et al. [10] addressed a future scenario of an approximately 2 °C increasinge, concluding that increase in monthly summer temperature should be expected to increase the duration of tularemia outbreaks in Sweden, and Ma et al. [6] showed generally high tularemia sensitivity to hydroclimatic variability and change.

In view of the quite limited investigations so far, and their different perspectives and conclusions, this study aims to more comprehensively consider future climate change projections and assess their implications for tularemia incidence. This is done with focus on change trends in disease outbreaks along the steep climatic gradient spanned by different Swedish sites (counties) with relevant data and previously established statistical disease models, which are here combined with the latest outputs of a multi-model ensemble of global climate models (GCMs) from phase six of the Coupled Model Intercomparison Project (CMIP; [18]).

## 2. Methods

### 2.1. Six High-Risk Counties with Established Statistical Models of Tularemia

**Figure 1:**
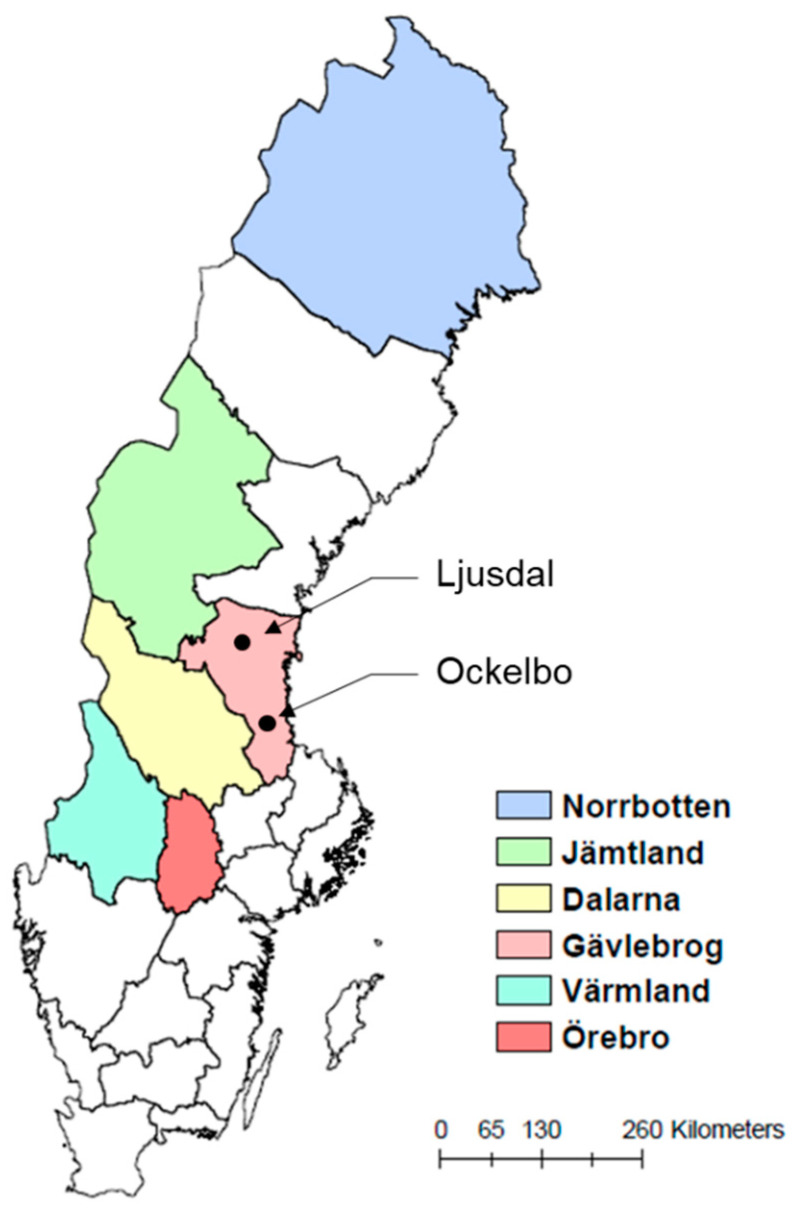
Six counties in Sweden, which fully or partly cover previously-identified high-risk sites for tularemia, and are the cases for future disease projections in the present study.

The cases considered for future disease trend projections are six Swedish counties (Norrbotten, Jämtland, Dalarna, Gävleborg, Värmland, and Örebro) distributed throughout Sweden (Figure 1). These counties fully or partly encompass seven previously identified tularemia high-risk regions that account for 56.4% of tularemia cases in Sweden, even though they contain only 9.3% of Sweden’s population, according to disease surveillance data for 1984–2012 [14]. Moreover, projection at county scale is consistent with the administration system in Sweden, where reported diseases are managed per county.

**Figure 2:**
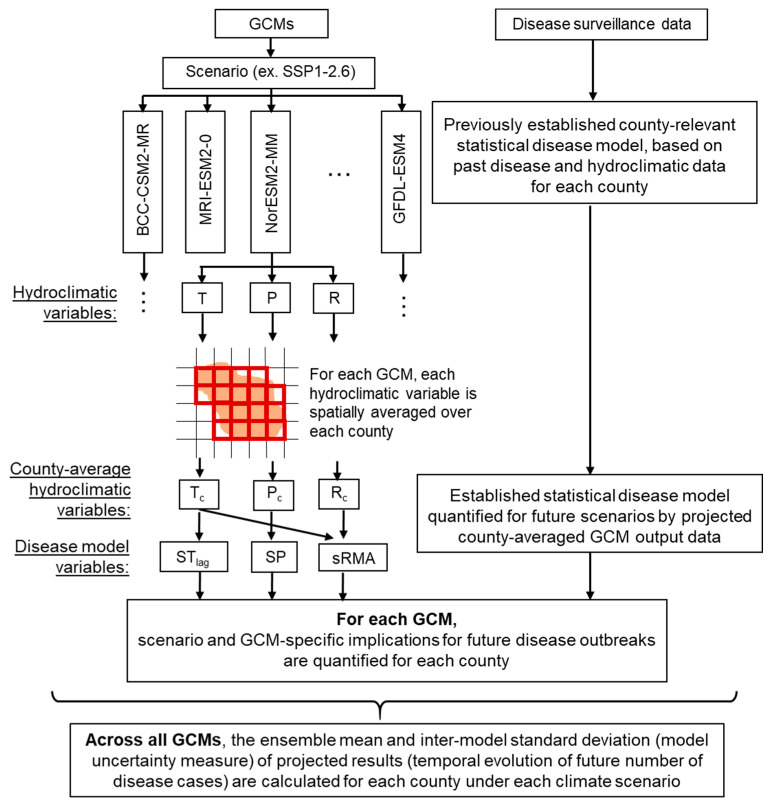
Schematic diagram of the model and statistical analysis approach in this study. For each county, we selected daily outputs of disease-relevant hydroclimatic variables (related to air temperature T, precipitation P, and runoff R) for the grid cells of each global climate model (GCM) with at least 40% of their area located within each targeted county (35% for Örebro county in The Geophysical Fluid Dynamics Laboratory’s Earth System Model version 4 (GFDL-ESM4) and The Geophysical Fluid Dynamics Laboratory’s climate model version 4 (GFDL-CM4)). We spatially averaged the values of each hydroclimatic output variable of each GCM over these county grid cells to represent a corresponding county-average daily hydroclimatic variable value (air temperature T_c_, precipitation P_c_, and runoff R_c_). Furthermore, for each GCM, we assessed its hydroclimatic projection implications for the future scenario evolution of disease cases in each county, based on a previously established county-relevant statistical disease model that we quantified by preceding-year number of disease cases and relevant GCM-projected county-average hydroclimatic variables (including summer temperature in the preceding year ST_lag_, summer precipitation in the same year SP, and standardized relative annual mosquito abundance sRMA, which is in turn related to various river flow Q conditions). Finally, across all GCMs, we calculated the model ensemble mean and inter-model standard deviation (as a GCM uncertainty measure) of scenario-implied possible future evolution of disease cases in each county. SSP1-2.6: Shared Socioeconomic Pathways (SSP) with emissions driven by sustainable practices to produce radiative forcing of 2.6 Wm^−2^ by 2100; BCC-CSM2-MR: The Beijing Climate Center Climate System Model, Version 2-MR; MRI-ESM2-0: The Meteorological Research Institute Earth System Model, Version 2.0; NorESM2-MM: The Norwegian Earth System Model, Version 2-MM.

Previously established statistical models (fitted to data) have been reported and are of the same type but with different coefficients for each Swedish site [14]. Statistical models do not assume known functional relationships between infectious disease spreading and hydroclimatic and other environmental variables, but quantify the data-given statistical signals of such relationships so far [19]. These can further be used to quantify the implications of GCM-projected scenarios of future hydroclimatic changes for diseases like tularemia, for which adequate mechanistic knowledge is still lacking, e.g., on bacteria ecology and transmission routes to humans [10]. Figure 2 illustrates schematically the overall approach to such quantification developed and applied in this study.

The disease models used in this study were selected because they are, so far, the only quantitative models available that have been developed and tested for modeling of temporal variations of tularemia cases in direct relation to associated hydroclimatic conditions, based on actual data for targeted high-risk regions in Sweden from 1984 to 2012 [14] . The basic disease model type is power-law scaling, which relates the annual number of tularemia cases (Tul) to the preceding-year number of cases (Tul_lag_) with exponent $\beta_{1}$, as Equation (1) [14,17]:

(1) $$\mathrm{Tul}=\mathrm{EXP}\left(\beta_{0}+\beta_{2}\mathrm{sRMA}+\beta_{3}{\mathrm{ST}}_{\mathrm{lag}}+\beta_{4}\mathrm{SP}\right)*{\mathrm{Tul}}_{\mathrm{lag}}{}^{\beta_{1}}=A*{\mathrm{Tul}}_{\mathrm{lag}}{}^{\beta_{1}}$$

The scale factor A in Equation (1) is determined by disease-independent hydroclimatic variables of: summer temperature in the preceding year (ST_lag_, °C); summer precipitation in the same year (SP, mm); along with standardized relative annual mosquito abundance (sRMA). The latter, derived from annual mosquito aboundance(RMA), in turn fully depends on hydroclimatic variables, as expressed by Equations (2)–(5) [14,17]:

(2) $$\mathrm{sRMA}=\frac{\log_{2}\mathrm{RMA}-\mathrm{mean}\left(\log_{2}\mathrm{RMA}\right)}{\mathrm{SD}\left(\log_{2}\mathrm{RMA}\right)}$$

(3) $$\mathrm{RMA}=\mathrm{Median}\left({\mathrm{RMA}}_{\left(t\right)}\right)$$

(4) $${\mathrm{RMA}}_{\left(t\right)}=2^{S_{N\left(F\right)}\mathrm{SM}}$$

(5) $$\mathrm{SM}=-2.76+0.67Q_{1}+0.62Q_{2}+0.19T$$

where mean and SD (standard deviation) denotes the sample mean and sample standard deviation of RMA; RMA is median value of daily mosquito aboundance ${\mathrm{RMA}}_{\left(t\right)}$; SM is the standardized mosquito abundance; $S_{N\left(F\right)}$ is the standard deviation of SM observed in the mosquito modeling area, which is cancelled out when substituted into Equation (2); Q1 and Q2 (unitless) are maximum standardized river flows in two time periods preceding each RMA evaluation time t (36–42 days and 22–28 days, respectively, before time t), and mean temperature (T, °C) over 1–7 days before time t. Original model expressions also included winter days with low snow coverage (CW, days) in A, but the variation in Tul has been shown to be insensitive to CW [6]; as such, it has been omitted from this analysis, for simplicity and increased clarity. Mosquitoes are main disease vectors in the large boreal forest regions of Alaska, Sweden, Finland, and Russia [20,21,22], and the statistical disease models used in this study include mosquito abundance as a main variable; in a fitted statistical model, however, such a variable may also be a proxy for other vectors.

Table 1 lists the $\beta_{0}$–$\beta_{4}$ coefficients in the power-law scaling Equation (1), as calculated and assigned to each Swedish county based on reported best fits to outbreak data for the associated high-risk sites [14,17]. For a high-risk site extending over more than one county, the associated site coefficient values were allocated to the county containing the largest proportion of the high-risk site. Gävleborg county contains two high-risk sites (Ockelbo, southern part of the county, and Ljusdal, northern part of the county) and was assigned two comparative sets of coefficients.

### 2.2. Climate Change Scenario Data, Human Tularemia Data, and Projection of Annual Cases

We used daily data for required hydroclimatic variables over 2015–2100 from GCMs that provide such outputs in phase six of the Coupled Model Intercomparison Project, CMIP6 (with raw output data downloaded from https://esgf-node.llnl.gov/projects/cmip6/). The required variables are air temperature T (corresponding to GCM variable: tas), precipitation P (pr), and runoff R (mrro; which in turn relates to river flow Q as R times the (constant) river catchment area). Because the finest available spatial resolution for daily runoff R is 100 km, we chose this spatial resolution also for the other hydroclimatic variables (T and P).

With regard to climate projection scenarios, we considered the low, medium, and high end scenarios of Shared Socioeconomic Pathways (SSPs) from the CMIP6 range of future radiation and emission pathways, i.e., SSP1-2.6, SSP2-4.5, and SSP5-8.5, respectively [23]. The SSP1-2.6 scenario represents low emissions driven by sustainable practices to produce radiative forcing of 2.6 Wm^−2^ by 2100, leading to a multi-model mean warming projection of significantly less than 2 °C warming by 2100, while the SSP5-8.5 scenario represents sufficiently high emissions to produce radiative forcing of 8.5 Wm^−2^ by 2100, leading to projected warming of 4.9 °C by 2100. The scenario SSP2-4.5 represents an intermediate radiative forcing level following continued historical patterns, leading to likely more realistic model projections than the other two more extreme scenarios. As such, we show in the following main results for the SSP2-4.5 climate scenario, and comparative results from the other two scenarios in Supplementary Material.

For each climate scenario, we only considered GCMs with daily outputs of all hydroclimatic variables required for full $\beta_{0}\;$–$\beta_{4}$ quantification, as listed in Table 2.

According to the overall study approach illustrated in Figure 2, we selected for each target county the relevant daily outputs for GCM grid cells with at least 40% of their area located within the county (35% for Örebro county in the Geophysical Fluid Dynamics Laboratory’s (GFDL) model GFDL-ESM4 and GFDL-CM4). We further spatially averaged the values of each output variable in these grid cells to represent a corresponding county-average daily value, and further quantified the associated Tul result for each GCM from the disease model Equation (1). Finally, we averaged the Tul results across all GCMs to determine and illustrate GCM-ensemble mean annual Tul values, and associated inter-model standard deviations (as a GCM uncertaintly measure) around the ensemble mean, for each county and for each considered climate scenario. In addition, we also determined ensemble mean values of each GCM-projected hydroclimatic and related disease variable included in the basic tularemia model Equation (1), in order to also separately illustrate the projected temporal evolutions of these county-average variables in each projected climate scenario. Initial values of Tul_lag_ for year 2015 were determined from data on human tularemia outbreaks for each county obtained from the data repository of the Nordic project CLINF [24] as listed in Supplementary Material Table S1.

### 2.3. Typology of Model Behavior Under Mean Climate State in the Long-Term

**Figure 3:**
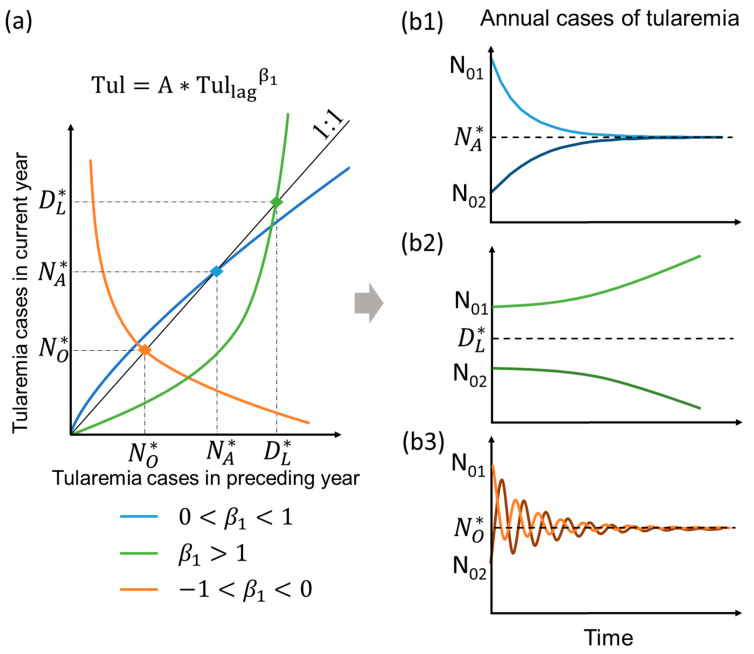
Conceptual diagram of: (**a**) different types of power-law scaling of number of tularemia outbreaks in a year (Tul) with the preceding-year number of outbreaks (Tul_lag_); and (**b1**–**b3**) the associated evolution of Tul with time. The curve intersections with the black 1:1 line at Tul = Tul_lag_ = $N_{A}^{*}$, $D_{L}^{*}$, $N_{O}^{*}$ in (**a**) represent endemic convergence level (dashed line in (**b1**)), divergence level (dashed line in (**b2**)), and endemic convergent oscillation level (dashed line in (**b3**)), respectively, for Tul after a shift in long-term average hydroclimatic conditions from a initial Tul_lag_ level larger (N_01_) or smaller (N_02_) than $N_{A}^{*}$, $D_{L}^{*}$, $N_{O}^{*}$ (i.e., in A of the best-fit power-law scaling $\mathrm{Tul}=A*{\mathrm{Tul}}_{\mathrm{lag}}{}^{\beta_{1}}$ for different exponent $\beta_{1}$ cases of relevance for the Swedish study counties (Table 1).

For any projected combination of hydroclimatic variable values determining the scale factor A, the power-law scaling of $\mathrm{Tul}=A*{\mathrm{Tul}}_{\mathrm{lag}}{}^{\beta_{1}}$ in Equation (1), with $\beta_{1}$ values given for the different Swedish counties in Table 1, follows the distinct types of behaviour illustrated in Figure 3a. Specifically, blue and green curves represent sublinear ($0\;<\;\beta_{1}<\;$1; most county cases, Table 1) and superlinear ($\beta_{1}$ > 1; Norrbotten case, Table 1) conditions of positive $\beta_{1}$, respectively, and the orange curve represents sublinear negative $\beta_{1}$ conditions ($-1\;<\;\beta_{1}<\;$0; Gävleborg (Ockelbo) case, Table 1). All three (power-law scaling) types of curves intersect some point of the black 1:1 line, which in turn represents (linear $\beta_{1}\approx 1$ conditions with) unchanging number of tularemia outbreaks over time (Tul = Tul_lag_).

For the most common sublinear positive case ($0\;<\beta_{1}<\;1)$, the power-law curve (blue in Figure 3a) intersects the 1:1 line at $N_{A}^{*}$, and implies convergence of Tul over time to this $N_{A}^{*}$ level, for any shift in long-term average hydroclimatic conditions (i.e., shift in A) and initial ${\mathrm{Tul}}_{\mathrm{lag}}$ level larger (N_01_) or smaller (N_02_) than $N_{A}^{*}$ (Figure 3b1); we refer to $N_{A}^{*}$ as the endemic convergence level, in consistency with the definition and use of this term also in Ma et al. (2019). In contrast, for the superlinear positive case ($\beta_{1}\;>\;1)$, the power-law curve (green in Figure 3a) intersects at $D_{L}^{*}$, and implies divergence of Tul over time to increasingly greater or smaller values than $D_{L}^{*}$, for any hydroclimatic shift in A and initial ${\mathrm{Tul}}_{\mathrm{lag}}$ level larger (N_01_) or smaller (N_02_) than $D_{L}^{*}$, respectively (Figure 3b2); we refer to $D_{L}^{*}$ as a divergence level for future tularemia outbreaks. For the sublinear negative case ($-1\;<\;\beta_{1}<\;0$), the power-law curve (orange in Figure 3a) intersects at $N_{O}^{*}$, and implies convergent oscillation of Tul (i.e., with decreasing oscillation amplitude) around $N_{O}^{*}$ over time, for any hydroclimatic shift in A and initial ${\mathrm{Tul}}_{\mathrm{lag}}$ level larger (N_01_) or smaller (N_02_) than $N_{O}^{*}$ (Figure 3b3); we refer to $N_{O}^{*}$ as endemic oscillation level. There is no such case among the Swedish study counties but, for the sake of completeness, we note that a superlinear negative case ($\beta_{1}<-1$) would imply divergent oscillation of Tul (i.e., with increasing oscillation amplitude) around a constant level analogous to $N_{O}^{*}$ for the sublinear negative case. Endemic convergence (for $0<\beta_{1}<1)$ and endemic oscillation (for $-1<\beta_{1}<0$) both imply an expected constant level toward/around which tularemia cases will tend to converge with time after a shift in long-term average hydroclimatic conditions (shift in A), so we combined these two terms into simply referring to the endemic level expected for any (positive or negative) sublinear power-law scaling of Tul with Tul_lag_.

## 3. Results

### 3.1. Change Trends for Hydroclimatic and Related Mosquito Abundance Variables

**Figure 4:**
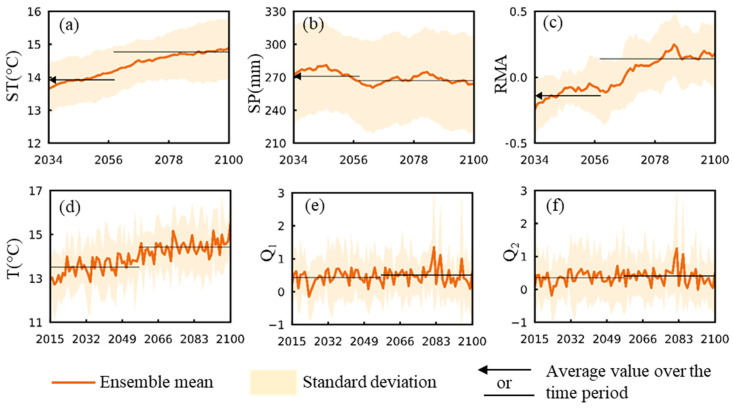
Projected ensemble mean results (orange line) ± 1 inter-model standard deviation (shaded areas) for the example of Dalarna county under the CMIP6 climate scenario SSP2-4.5. (**a**) Summer temperature (ST, °C), (**b**) summer precipitation (SP, mm), (**c**) standardized relative mosquito abundance (sRMA), (**d**) mean temperature (T) over 1–7 days before time t of the sRMA evaluation, along with maximum standardized river flows (**e**) Q_1_ and (**f**) Q_2_ over 36–42 days and 22–28 days before t, respectively. Results are shown in terms of 20–year running averages in (**a**–**c**), and in terms of annual average values in (**d**–**f**). Black lines show average values over the time periods 2015–2057 and 2058–2100. Supplementary Figures S1 and S2 show results for all counties corresponding to panels (**a**–**c**) and (**d**–**f**), respectively.

Figure 4 shows Dalarna county as an example for the resulting GCM ensemble mean time series (solid lines) and corresponding inter-GCM ± 1 standard deviation (shaded area) of (a) yearly summer temperature (ST), (b) summer precipitation (SP), (c) standardized relative mosquito abundance (sRMA), (d) mean temperature (T) over 1–7 days before time t of the sRMA evaluation, along with maximum standardized river flows (e) Q_1_ and (f) Q_2_ over 36–42 days and 22–28 days before t, respectively, for climate scenario SSP2-4.5. Results are shown in terms of 20-year running averages in (a–c), and in terms of annual average values in (d–f). Black lines show average values over the time periods 2015–2057 and 2058–2100. Supplementary Figures S1 and S2 show results for all counties corresponding to panels (a–c) and (d–f), respectively. For comparison, corresponding results of T, SP, and sRMA for the other two climate scenarios are also shown in Supplementary Material Figure S3, with the 43-year averages for the two climate periods 2015–2057 and 2058–2100 also shown in histograms in Supplementary Material Figure S4.

For climate scenario SSP2-4.5, all counties exhibit similar weakly increasing trends for ST (Figure 4a, Figure S1a), with scenario SSP1-2.6 yielding even weaker and scenario SSP5-8.5 leading to steeper increase trends (Figure S3a). For SP, change trends are overall small for all counties and climate scenarios (Figure 4b, Figure S1b, Figure S3b). For sRMA in scenario SSP2-4.5, all counties have similar subtle increase trends (Figure 4c, Figure S1c), which also applies to the other two climate scenarios (Figure S3c), with the exception of Örebro county for SSP1-2.6. Overall, similar inter-GCM uncertainty levels are exhibited for these three hydroclimatically dependent variables among counties and climate scenarios. Figure 4d–f and Figure S2 further show projected ensemble mean results for scenario SSP2-4.5 of the three hydroclimatic variables underlying and determining sRMA from 2015 to 2100. Values of temperature T generally increase while values of standardised flow Q_1_ and Q_2_ exhibit mainly small-range fluctuations over time.

### 3.2. Tularemia Change Trends Under Projected Future Hydroclimatic Conditions

**Figure 5:**
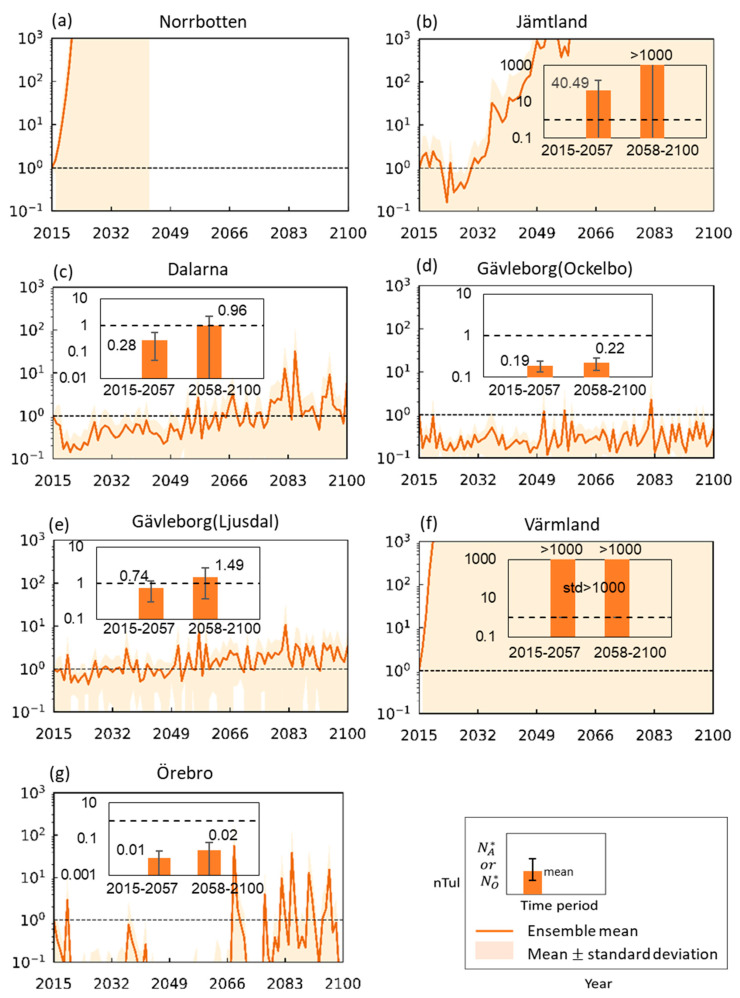
Normalized projected ensemble mean number of annual tularemia cases (nTul) for 2015–2100, and (inserts) associated normalized endemic levels ($N_{A}^{*}$ or $N_{O}^{*}$ ) for the periods 2015–2057 and 2058–2100. Results are shown for the Swedish counties Norbotten (**a**), Jämtland (**b**), Dalarna (**c**), Gävleborg (two sites) ((**d**) and (**e**)), Värmland (**f**), and Örebro (**g**) under CMIP6 climate scenario SSP2-4.5. The shaded areas (main figures) and error bars (inserts) show ± 1 inter-model standard deviation around the model ensemble mean.

Figure 5 shows resulting projected ensemble means of annual tularemia cases for scenario SSP2-4.5 for each county, along with associated endemic levels (Insert figures; expected divergence level for Norrbotten is shown in Supplementary Material Figure S5) over two climate periods (2015–2057 and 2058–2100). Comparison with the other two climate scenarios is shown in Supplementary Material Figure S5. All values shown are normalized with the respective actual numbers of tularemia cases occurring in year 2015 in each county (listed explicitly in Supplementary Table S1). The normalization focuses on and facilitates direct comparability of overall change trends and associated uncertainty levels for and across different counties. Furthermore, it avoids giving a misleading impression of predicting absolute numbers of disease cases at specific future points in time. Even though change trends are relatively mild and largely similar among counties for the hydroclimatic variables determing the scale factor A in the disease Equation (1), the counties exhibit widely diverging resulting tularemia change patterns, with consistent trends in endemic levels.

Annual disease cases in Norrbotten are projected to surge towards much higher levels if no counter-measures are taken. Large and rapid disease increases also emerge for Värmland and Jämtland. In contrast, projected annual cases in Dalarna and Gävleborg (Ljusdal) show overall slower and smaller increase trends, while Gävleborg (Ockelbo) and Örebro exhibit overall and intermittent disease decreases (values <1) from the 2015 disease status.

Comparison with the other two climate scenarios (Figure S5) shows similar dramatic rise trends for Norrbotten, Värmland, and Jämtland, while Dalarna and Gävleborg (both sites) exhibit increase in endemic levels with higher scenario forcing level. Örebro exhibits mixed annual case occurrence among climate scenarios and lowest endemic level for the intermediate scenario SSP2-4.5, but overall projected endemic levels for Örebro are <1 (i.e., smaller than the 2015 number of cases) and near-zero across all scenarios. Inter-model uncertainties can be large, and are particularly dramatic for Norrbotten, Jämtland and Värmland, while they are relatively small for Gävleborg (Ockelbo).

## 4. Discussion and Conclusions

Results for the six Swedish counties show large tularemia sensitivity to relatively small hydroclimatic change trends, and large inter-GCM uncertainty levels for disease projections compared to those for the underlying hydroclimatic variables. High sensitivity to the power-law disease scaling characteristics is also evident in the widely different disease projection results under more or less similar hydroclimatic change trends among the counties. Among counties, the relatively southern counties of Örebro and Gävleborg (Ockelbo) exhibit periodic or overall tularemia declines, respectively, while the most northern counties Norrbotten and Jämtland, but also the southern Värmland, exhibit large increases, and the intermediate Dalarna and Gävleborg (Ljusdal) exhibit intermediate trends of mostly increases (depending on climate scenario) until 2100.

In general, projected long-term trends in endemic levels are closely related to the power-law exponent $\beta_{1}$ for scaling of Tul with Tul_lag_. With Norrbotten having superlinear $\beta_{1}>\;1$ and Värmland and Jämtland having sub- but near-linear $\beta_{1}$ = 0.99 and 0.93, respectively, associated tularemia and endemic level impacts tend to be enhanced by projected shifts in hydroclimatic (scale factor A) conditions. The differences in projected tularemia cases among counties and some mixed results for the three climate scenarios challenge possible notions that climate change (and higher emission scenarios) will generally lead to (higher) increases of disease incidence. The different best-fit $\beta_{1}$ values for the different counties may then implicitly reflect geographic differences in, e.g.,: (1) demographics; (2) risk of pathogen exposure; (3) other local conditions/measures affecting vulnerabity/resilience to disease; (4) and interactions among hydroclimatic factors. This notion has also been discussed in other research using statistical disease modeling to project future disease burden under various climate scenarios. For example, Hales et al. (2002) [25] estimated that climate change would lead to 50–60% of the global population being at risk of dengue transmission by 2085, compared with 35% without the projected climate change. For falciparum malaria, however, Rogers and Randolph (2000) [26] projected that, by 2050, 23 million hosts would be gained in previously uninfected regions while 25 million human hosts would be lost in areas no longer suitable for transmission, which would lead to little net disease change in total.

With regard to the latter, the high-emissions scenario (SSP5-8.5) led to similar increases in summer temperature across the counties, but warming-related changes in precipitation and runoff affected tularemia results most. Other studies have found that human-related factors may play a more important disease role than climate, e.g., superior healthcare infrastructure might lead to net lowering of disease impacts even if climate change enhances pathogen ranges [19]. The history of widely studied diseases (e.g., malaria, yellow fever, dengue fever) also shows that human activities and their impacts on local ecology may affect disease spreading more significantly than climate change [27]. In addition to external drivers, internal complexity of climate-disease interactions also affects disease risk. For example, an increase in temperature may increase mosquito biting rates, parasite replication within mosquitoes, and mosquito development, but also increase mosquito mortality, making disease outcomes difficult to determine [28].

This study also has several limitations. First, in Sweden, high tularemia incidence usually appears in low- population areas [14]. So, although an area can have high outbreak rate projections (such as Norrbotten and Värmland in this study), local population levels may set lower upper limits for outbreaks. Second, the considered disease models do not explicitly take human behavioral factors into account, such as time spent on outdoor activities, which is an important factor for exposure of humans to the disease. Moreover, the statistical tularemia models have been rather inaccurate in estimating the magnitude of recent outbreaks, especially for the two most northern counties (Norrbotten and Jämtland) [14]. Thus, the present results for how hydroclimatic changes may impact future outbreaks should be used with caution, as comparative indications rather than in terms of absolute disease outbreak projections.

Uncertainties are inevitable in and among projected results of different climate models, and in all studies using climate model outputs in other types of models. The latter, propagated type of climate-related uncertainties are here shown to be amplified in disease models with high climate sensitivity, such as the tularemia models for Norrbotten, Jämtland, and Värmland in this study. Bring et al. (2019) [29] found relatively good model-data agreement for ensemble mean outputs of runoff and temperature in the Nordic-Arctic region, but worse agreement for precipitation outputs, and considerable doubt still remains about realism and accuracy of hydroclimatic results from individual GCMs. Contradictions inevitably emerge in disease projections for localized transmission routes [14] and future work needs to continue exploration of opportunities to improve projection realism and accuracy.

Well-archived data of infectious diseases in clinics and laboratories, along with adequately-recorded climate, hydrological, and other environmental as well as socio-economic data in recent years has made it feasible to develop statistical disease models, which can further be used in combination with related projected hydroclimatic and other types of data to quantify scenarios of possible future disease evolution (Figure 2). The available historical records along with forthcoming new scenario-projected model data and model-coupling methods will surely benefit more accurate large-scale assessments of future disease pressures and risks. In addition, advancements in mechanistic disease modeling are needed to bridge gaps and overcome weakness of statistical models, which may not be as relevant for other locations and new hydroclimatic and other environmental and societal conditions than the ones they were fitted to.

In conclusion, this study has quantified the implications of scenario-projected future hydroclimatic trends for possible future disease evolution, using site-specific, established, and parameterized statistical tularemia models. Results show highly divergent disease change trends and fluctuation levels around these for future climate change scenarios among Swedish counties, with scenarios of steeper future climate warming not necessarily leading to steeper disease increases. The directions of future tularemia change trends are robust in some counties, as seen from results across various future climate scenarios and their representations by different GCMs. Such robust change-trend projections are essential to identify and useful in pointing out needs for policy and management measures to avoid clear negative directions of future disease evolution, even though uncertainties about absolute future disease numbers may be large.

## Figures and Tables

**Table 1:** Tularemia model coefficients assigned to the six Swedish study counties.

High-Risk County	$\mathrm{Intercept}\;(\beta_{0})$	${\mathrm{Tul}}_{\mathrm{lag}}\;(\beta_{1})$	$\mathrm{sRMA}\;(\beta_{2})$	${\mathrm{ST}}_{\mathrm{lag}}\;(\beta_{3})$	$\mathrm{SP}\;(\beta_{4})$
Norrbotten	−5.73	1.16	0.20	0.35	0.005
Jämtland	−11.47	0.93	0.75	0.84	0.003
Gävleborg (Ockelbo)	−2.86	−0.19	0.36	0.10	0.010
Gävleborg (Ljusdal)	−7.60	0.09	0.29	0.50	0.009
Dalarna	−10.74	0.37	0.43	0.67	0.008
Värmland	10.11	0.99	0.82	−0.39	−0.014
Örebro	−9.19	0.73	0.75	0.18	0.023

β: coefficients in the power-law scaling Equation (1); Tul_lag:_ the annual number of tularemia cases to the preceding-year number of cases; sRMA: standardized relative annual mosquito abundance; ST_lag_: summer temperature in the preceding year; SP: summer precipitation in the same year.

**Table 2:** List of global climate models (GCMs) considered in this study.

Model	SSP1-2.6	SSP2-4.5	SSP5-8.5
BCC-CSM2-MR	X	X	X
MRI-ESM2-0	X	X	X
NorESM2-MM	X	X	X
EC-Earth3	X	X	X
* INM-CM5-0	X	X	X
* INM-CM4-8	X	X	X
MPI-ESM1-2-HR	X	X	X
GFDL-ESM4	X	X	X
GFDL-CM4		X	X

* Not included in Gävleborg county because of data error. Remarks: BCC-CSM2-MR: The Beijing Climate Center Climate System Model, Version 2-MR; MRI-ESM2-0: The Meteorological Research Institute Earth System Model, Version 2.0; NorESM2-MM: The Norwegian Earth; System Model, Version 2-MM; EC-Earth3: The European Consortium Earth System Model, Version 3; INM; CM5-0: The Institute for Numerical Mathematics Climate Model, version 5.0; INM-CM4-8: The Institute for; Numerical Mathematics Climate Model, Version 4.8; MPI-ESM1-2-HR: Max Planck Institute Earth System Model, Version 1.2-HR; GFDL-ESM4: The Geophysical Fluid Dynamics Laboratory’s Earth System Model version 4; GFDL-CM4: The Geophysical Fluid Dynamics Laboratory’s climate model version 4. SSP1-2.6: Shared Socioeconomic Pathways (SSP) with emissions driven by sustainable practices to produce radiative forcing of 2.6 Wm^−2^ by 2100; SSP2-4.5: Shared Socioeconomic Pathways with an intermediate radiative forcing level following continued historical patterns of 4.5 Wm^−2^ by 2100; SSP5-8.5: Shared Socioeconomic Pathways with sufficiently high emissions to produce radiative forcing of 8.5 Wm^−2^ by 2100.

## Supplementary Materials

The following are available online at https://www.mdpi.com/1660-4601/17/18/6786/s1, Figure S1: Projected ensemble mean results ± 1 inter-model standard deviation of relevant variables for each county under the CMIP6 climate scenario SSP2-4.5, Figure S2: Projected ensemble mean results ± 1 inter-model standard deviation of mosquito abundance dependent variables for each county under the CMIP6 climate scenario SSP2-4.5, Figure S3: Projected ensemble means of summer temperature, summer precipitation, and standardized relative mosquito abundance under three targeted scenarios, Figure S4: Forty-three-year mean of projected summer precipitation, summer precipitation, and standardized relative mosquito abundance under three targeted scenarios, Figure S5: Normalized projected annual tularemia cases (nTul) 2015-2100 (left panel) and normalized divergence or endemic levels in the periods 2015-2057 and 2058-2100; Table S1: Number of tularemia outbreaks in humans occurring in each county in year 2015.

## References

[1] Baker-Austin C., Trinanes J.A., Taylor N.G.H., Hartnell R., Siitonen A., Martinez-Urtaza J. (2013). Emerging Vibrio risk at high latitudes in response to ocean warming. Nat. Clim. Chang..

[2] Burge C.A., Mark Eakin C., Friedman C.S., Froelich B., Hershberger P.K., Hofmann E.E., Petes L.E., Prager K.C., Weil E., Willis B.L. (2014). Climate change influences on marine infectious diseases: Implications for management and society. Ann. Rev. Mar. Sci..

[3] Garrett K.A., Dobson A.D.M., Kroschel J., Natarajan B., Orlandini S., Tonnang H.E.Z., Valdivia C. (2013). The effects of climate variability and the color of weather time series on agricultural diseases and pests, and on decisions for their management. Agric. For. Meteorol..

[4] Harvell D., Altizer S., Cattadori I.M., Harrington L., Weil E. (2009). Climate change and wildlife diseases: When does the host matter the most?. Ecology.

[5] Rodó X., Pascual M., Doblas-Reyes F.J., Gershunov A., Stone D.A., Giorgi F., Hudson P.J., Kinter J., Rodríguez-Arias M.-À., Stenseth N.C. (2013). Climate change and infectious diseases: Can we meet the needs for better prediction?. Clim. Chang..

[6] Ma Y., Bring A., Kalantari Z., Destouni G. (2019). Potential for Hydroclimatically Driven Shifts in Infectious Disease Outbreaks: The Case of Tularemia in High-Latitude Regions. Int. J. Environ. Res. Public Health..

[7] Waits A., Emelyanova A., Oksanen A., Abass K., Rautio A. (2018). Human infectious diseases and the changing climate in the Arctic. Environ. Int..

[8] Malkhazova S., Mironova V., Shartova N., Orlov D. (2019). Mapping Russia’s Natural Focal Diseases: History and Contemporary Approaches.

[9] Petersen J.M., Mead P.S., Schriefer M.E. (2009). Francisella tularensis: An arthropod-borne pathogen. Vet. Res..

[10] Rydén P., Sjöstedt A., Johansson A. (2009). Effects of climate change on tularaemia disease activity in Sweden. Glob. Health Action.

[11] Hestvik G., Warns-Petit E., Smith L.A., Fox N.J., Uhlhorn H., Artois M., Hannant D., Hutchings M.R., Mattsson R., Yon L. (2015). The status of tularemia in Europe in a one-health context: A review. Epidemiol. Infect..

[12] Desvars A., Furberg M., Hjertqvist M., Vidman L., Sjöstedt A., Rydén P., Johansson A. (2015). Epidemiology and Ecology of Tularemia in Sweden, 1984–2012. Emerg. Infect. Dis..

[13] Balci E., Borlu A., Kilic A.U., Demiraslan H., Oksuzkaya A., Doganay M. (2014). Tularemia outbreaks in Kayseri, Turkey: An evaluation of the effect of climate change and climate variability on tularemia outbreaks. J. Infect. Public Health.

[14] Desvars-Larrive A., Liu X., Hjertqvist M., Sjöstedt A., Johansson A., Rydén P. (2017). High-risk regions and outbreak modelling of tularemia in humans. Epidemiol. Infect..

[15] Nakazawa Y., Williams R., Peterson A.T., Mead P., Staples E., Gage K.L. (2007). Climate Change Effects on Plague and Tularemia in the United States. Vector Borne Zoonotic Dis..

[16] Palo T.R., Ahlm C., Tärnvik A. (2005). Climate variability reveals complex events for tularaemia dynamics in man and mammals. Ecol. Soc..

[17] Rydén P., Björk R., Schäfer M.L., Lundström J.O., Petersén B., Lindblom A., Forsman M., Sjöstedt A., Johansson A. (2012). Outbreaks of Tularemia in a Boreal Forest Region Depends on Mosquito Prevalence. J. Infect. Dis..

[18] Eyring V., Bony S., Meehl G.A., Senior C.A., Stevens B., Stouffer R.J., Taylor K.E. (2016). Overview of the Coupled Model Intercomparison Project Phase 6 (CMIP6) experimental design and organization. Geosci. Model. Dev..

[19] Lafferty K.D. (2009). The ecology of climate change and infectious diseases. Ecology.

[20] Eisen R.J., Mead P.S., Meyer A.M., Pfaff L.E., Bradley K.K., Eisen L. (2008). Ecoepidemiology of Tularemia in the Southcentral United States. Am. J. Trop. Med. Hyg..

[21] Eliasson H., Lindbäck J., Nuorti J.P., Arneborn M., Giesecke J., Tegnell A. (2002). The 2000 Tularemia Outbreak: A Case-Control Study of Risk Factors in Disease-Endemic and Emergent Areas, Sweden. Emerg. Infect. Dis..

[22] Keim P., Johansson A., Wagner D.M. (2007). Molecular Epidemiology, Evolution, and Ecology of Francisella. Ann. N. Y. Acad. Sci..

[23] Gidden M.J., Riahi K., Smith S.J., Fujimori S., Luderer G., Kriegler E., van Vuuren D.P., van den Berg M., Feng L., Klein D. (2019). Global emissions pathways under different socioeconomic scenarios for use in CMIP6: A dataset of harmonized emissions trajectories through the end of the century. Geosci. Model Dev..

[24] CLINF Project—Climate Change Effects on the Epidemiology of Infectious Diseases and the Impacts on Northern Societies, 2020. The CLINF Data Repository, WP3: Depicting the Geographic Spread of Climate-Sensitive Infections in the Nordic Region. https://clinf.org.

[25] Hales S., de Wet N., Maindonald J., Woodward A. (2002). Potential effect of population and climate changes on global distribution of dengue fever: An empirical model. Lancet.

[26] Rogers D.J., Randolph S.E. (2000). The Global Spread of Malaria in a Future, Warmer World. Science.

[27] Reiter P. (2001). Climate change and mosquito-borne disease. Environ. Health Perspect..

[28] Rohr J.R., Dobson A.P., Johnson P.T.J., Kilpatrick A.M., Paull S.H., Raffel T.R., Ruiz-Moreno D., Thomas M.B. (2011). Frontiers in climate change–disease research. Trends Ecol. Evol..

[29] Bring A., Goldenberg R., Kalantari Z., Prieto C., Ma Y., Jarsjö J., Destouni G. (2019). Contrasting Hydroclimatic Model-Data Agreements Over the Nordic-Arctic Region. Earth’s Future.

