# Supplementary material for: Implications of Projected Hydroclimatic Change for Tularemia Outbreaks in High-Risk Areas across Sweden
Source: Int J Environ Res Public Health. 2020 Sep 17;17(18):6786. doi: 10.3390/ijerph17186786 (PMC7558863; doi:10.3390/ijerph17186786)
Supplement: Supplementary file 1 [file ijerph-17-06786-s001.pdf]

International Journal of
Environmental Research and Public Health

Supplementary material

Implications of Projected Hydroclimatic Change for Tularemia Outbreaks in High-Risk Areas Across Sweden

Table 1. Number of tularemia outbreaks in humans occurring in each county in year 2015.

County	Norrbotten	Jämtland	Dalarna	Gävleborg	Värmland	Örebro
Number of cases	135	6	16	11	17	7

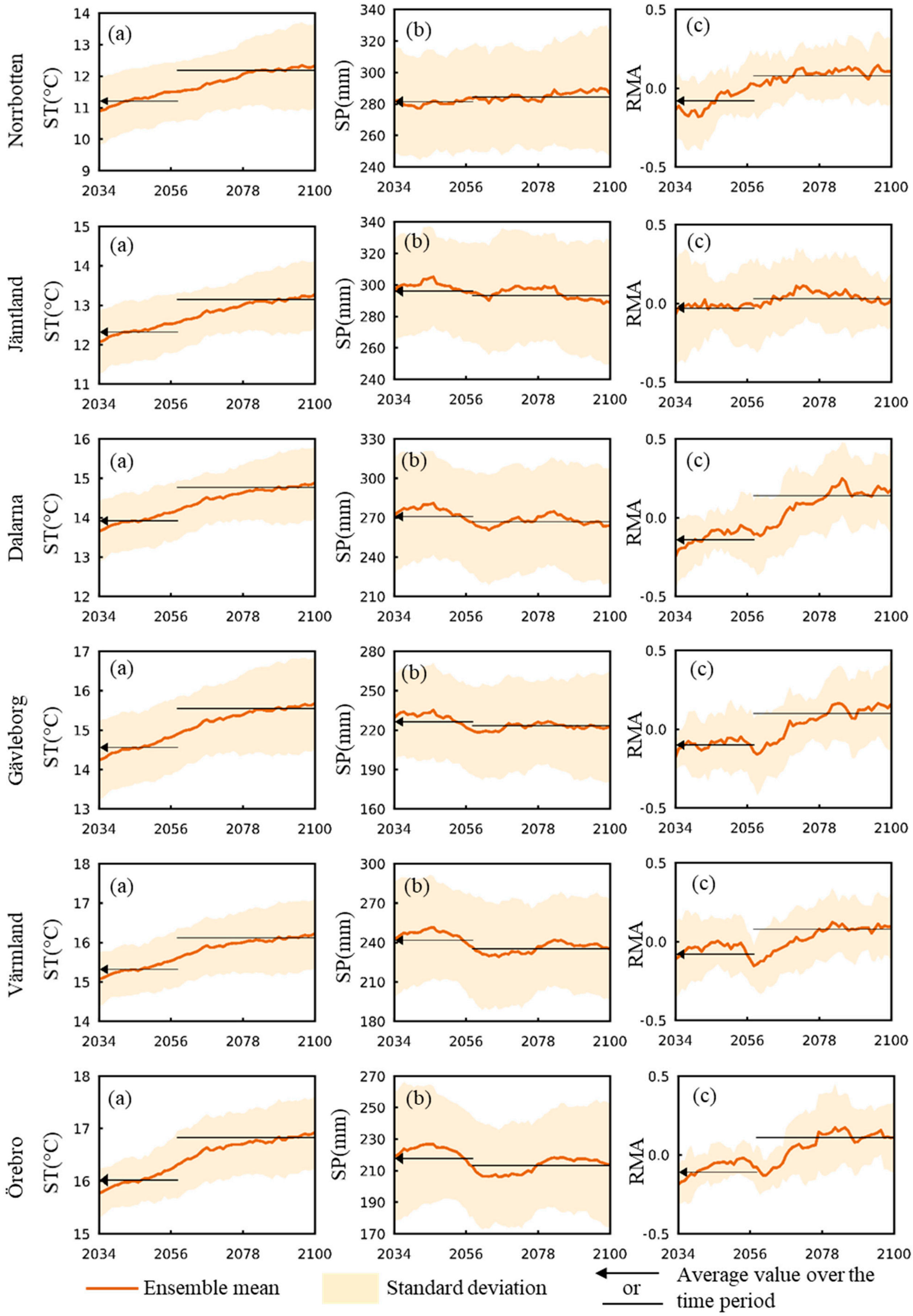
Norrbotten
Jämtland
Dalarna
Gävleborg
Värmland
Örebro
(a)
(b)
(c)
ST(°C)
SP(mm)
RMA
2034
2056
2078
2100
Ensemble mean
Standard deviation
or
Average value over the time period

Figure 1. Projected ensemble mean results (orange line) ± 1 inter-model standard deviation (shaded areas) for each county under the CMIP6 climate scenario SSP2-4.5. (a) Summer temperature (ST, °C), (b) summer precipitation (SP, mm), (c) standardized relative mosquito abundance (sRMA). Results are shown in terms of 20-year running averages. Black lines (or black lines with arrows) show average values over the time periods 2015-2057 and 2058-2100.

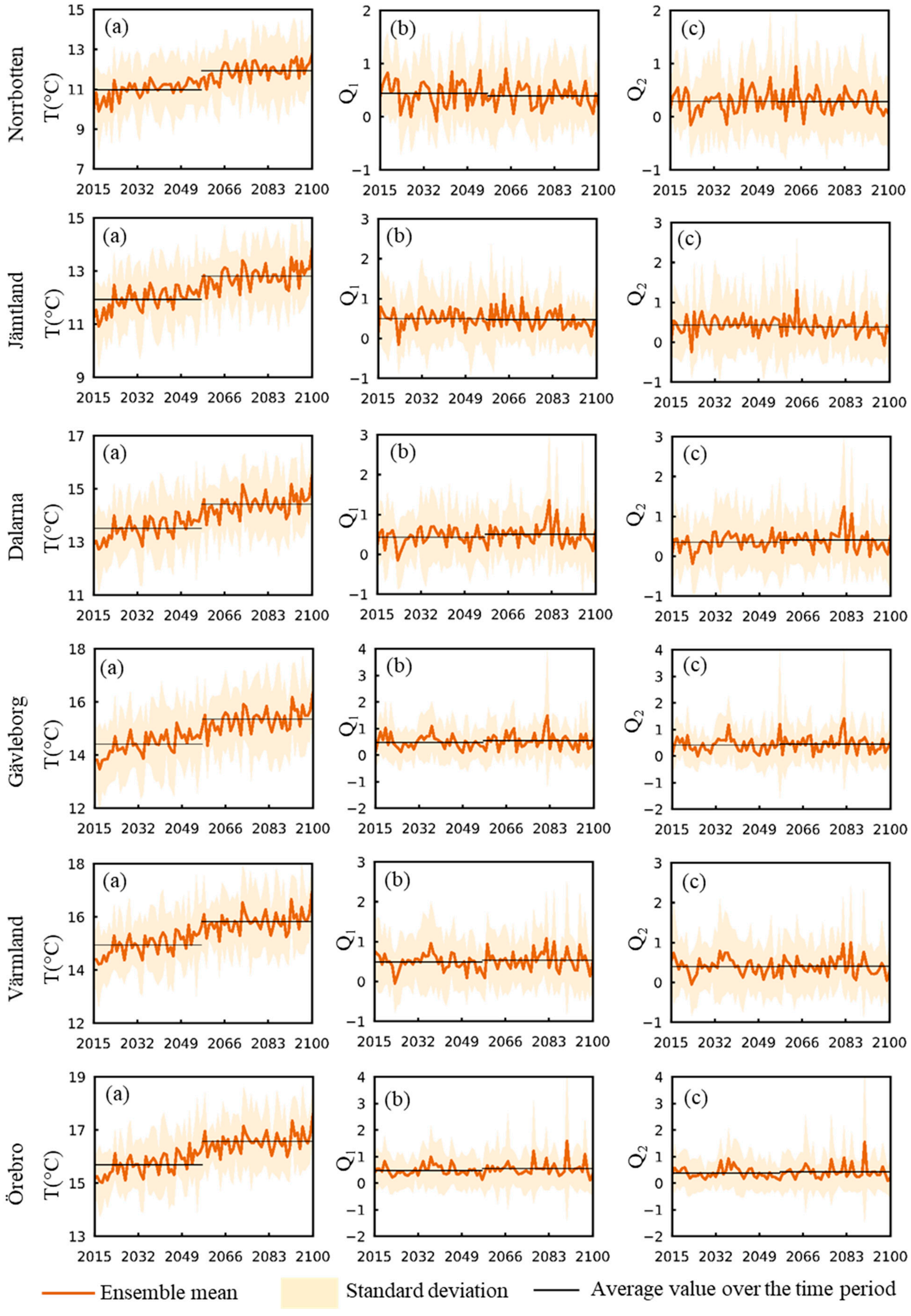

Figure 2. Projected ensemble mean results (orange line) ± 1 inter-model standard deviation (shaded areas) for each county under the CMIP6 climate scenario SSP2-4.5. (a) mean temperature (T) over 1-7

days before time t of the sRMA evaluation, along with maximum standardized river flows (b) Q1 and (c) Q2 over 36-42 days and 22-28 days before t, respectively. Results are shown in terms of annual average values. Black lines show average values over the time periods 2015-2057 and 2058-2100.

	**(a) Summer temperature, ST (°C)**	**(b) Summer precipitation, SP (mm)**	**(c) Standardized relative mosquito abundance, sRMA**

Norrbotten

(a) Summer temperature, ST (°C)

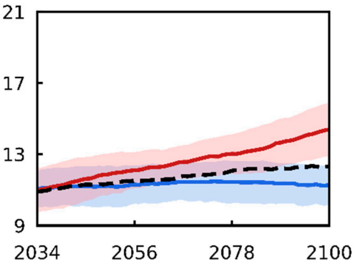

2015-2057	2058-2100
11.16	11.36
11.21	12.19
11.56	13.66

(b) Summer precipitation, SP (mm)

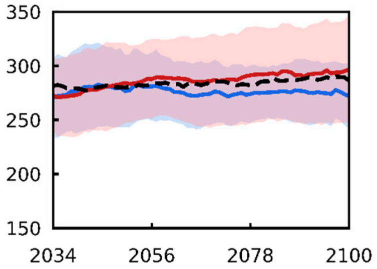

2015-2057	2058-2100
276.03	272.81
281.41	284.38
279.30	293.02

(c) Standardized relative mosquito abundance, sRMA

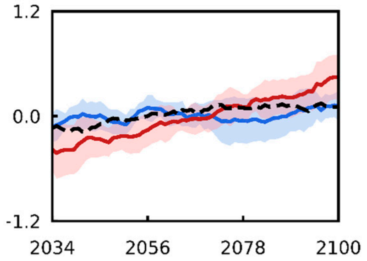

2015-2057	2058-2100
-0.02	0.02
-0.08	0.08
-0.28	0.28

Jämtland

(a) Summer temperature, ST (°C)

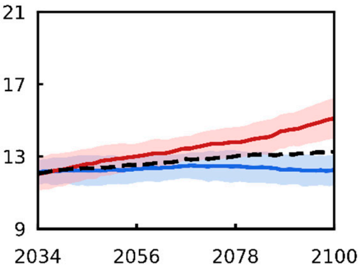

2015-2057	2058-2100
12.21	12.35
12.32	13.15
12.55	14.41

(b) Summer precipitation, SP (mm)

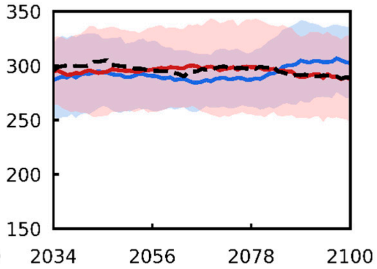

2015-2057	2058-2100
288.96	294.48
296.13	293.21
294.73	294.21

(c) Standardized relative mosquito abundance, sRMA

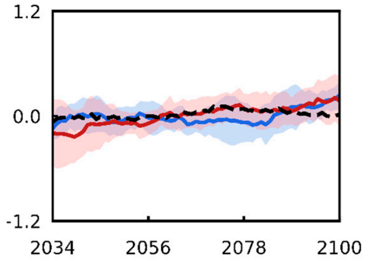

2015-2057	2058-2100
-0.06	0.06
-0.03	0.03
-0.15	0.15

Dalarna

(a) Summer temperature, ST (°C)

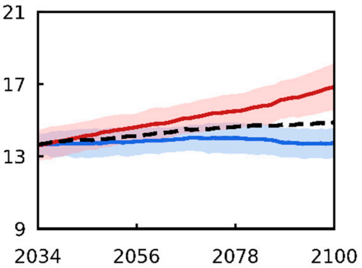

2015-2057	2058-2100
13.72	13.87
13.92	14.77
14.15	16.13

(b) Summer precipitation, SP (mm)

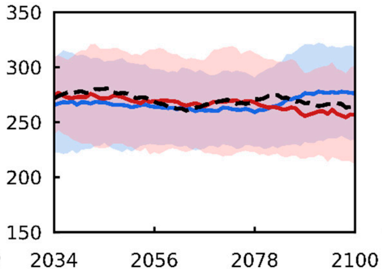

2015-2057	2058-2100
265.13	268.61
270.94	266.90
271.50	261.96

(c) Standardized relative mosquito abundance, sRMA

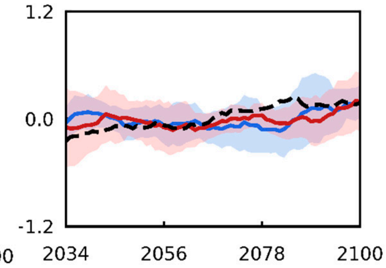

2015-2057	2058-2100
-0.04	0.04
-0.14	0.14
-0.10	0.10

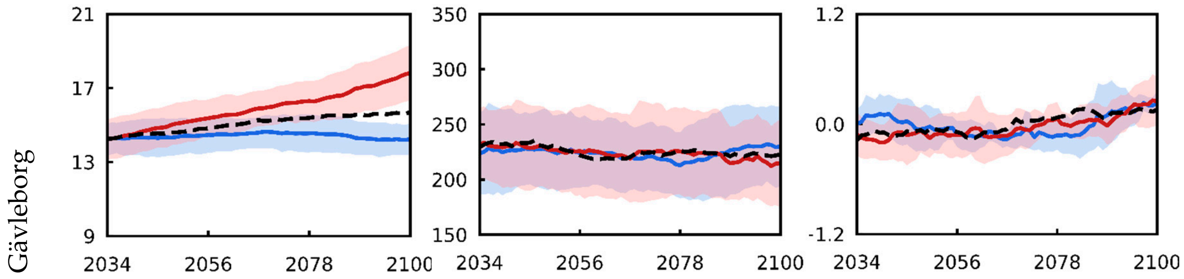

2015-2057	2058-2100
14.35	14.39
14.56	15.55
14.83	17.00

2015-2057	2058-2100
225.26	222.62
226.36	223.34
228.25	220.34

2015-2057	2058-2100
-0.04	0.04
-0.10	0.10
-0.14	0.14

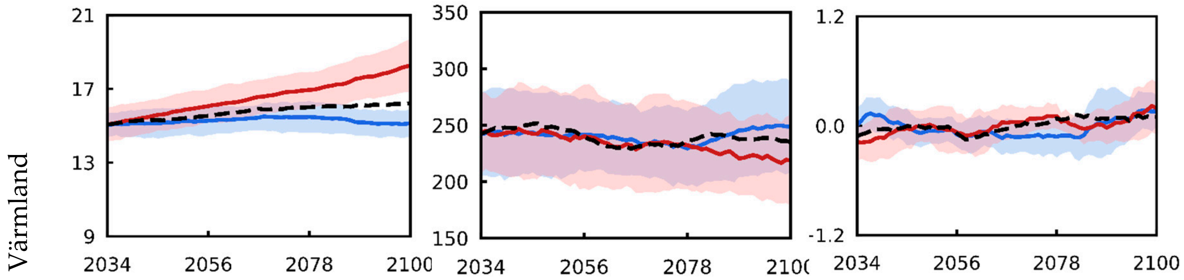

2015-2057	2058-2100
15.15	15.30
15.32	16.12
15.60	17.56

2015-2057	2058-2100
242.03	239.94
241.81	235.34
241.06	225.25

2015-2057	2058-2100
-0.02	0.02
-0.08	0.08
-0.13	0.13

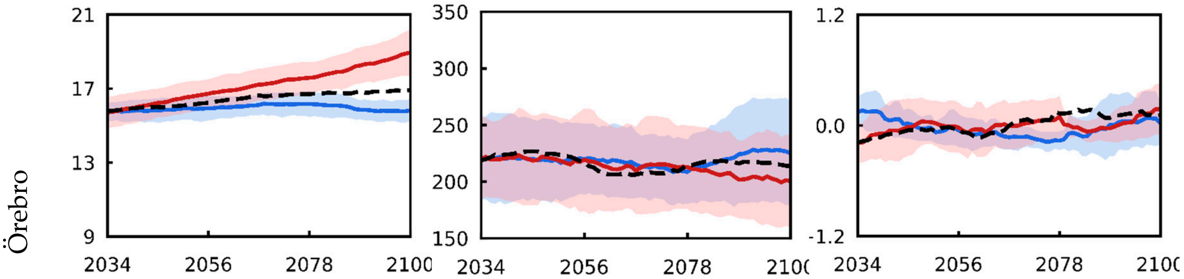

2015-2057	2058-2100
15.83	15.98
16.02	16.83
16.25	18.23

2015-2057	2058-2100
220.39	218.30
217.72	213.24
218.72	206.32

2015-2057	2058-2100
0.04	-0.04
-0.11	0.11
-0.10	0.10

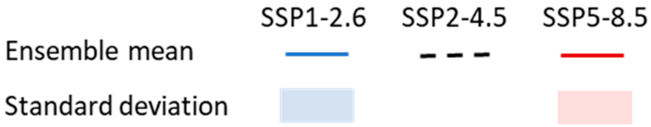

Figure 3. Projected ensemble means of summer temperature (ST, °C), summer precipitation (SP, mm), and standardized relative mosquito abundance (sRMA) in Norrbotten, Jämtland, Dalarna, Gävleborg, Värmland, Örebro county from 2034 to 2100 for scenario SSP1-2.6, SSP2-4.5, and SSP5-8.5 from CMIP6 (deviations from 20-year running mean). Shaded areas are ranges between mean ± standared

deviations. The tables underneath list 43-year average ST, SP, and sRMA of each variable of each county.

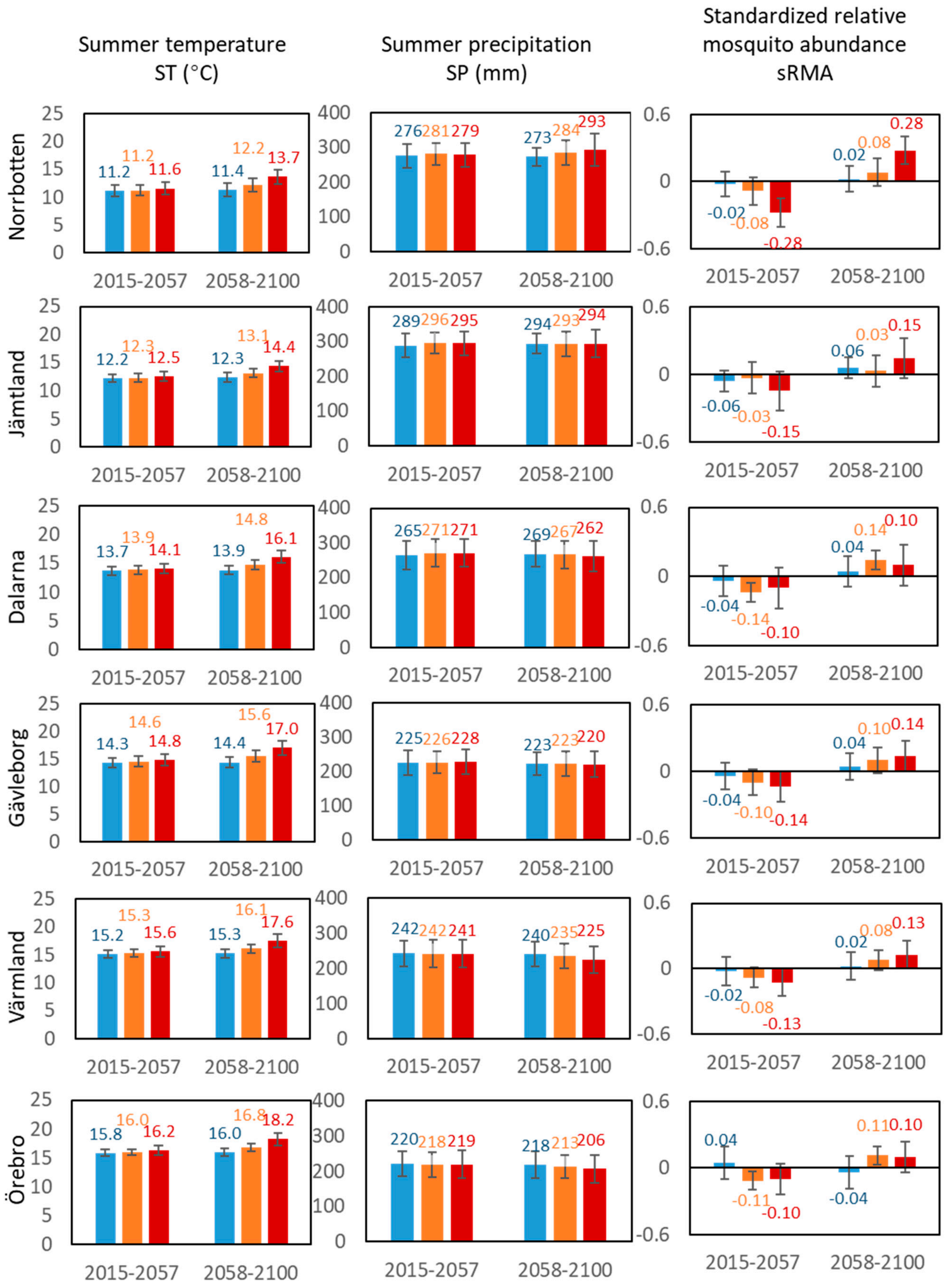

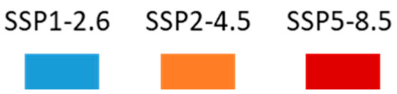

Figure 4. Forty-three-year mean of projected summer precipitation (ST), summer precipitation (SP), and standardized relative mosquito abundance (sRMA) for Norrbotten, Jämtland, Dalarna, Gävleborg, Värmland, and Örebro county in scenarios SSP1-2.6, SSP2-4.5 and SSP5-8.5 from CMIP6.

	Projected annual cases	Projected endemic levels and divergence levels
Norrbotten	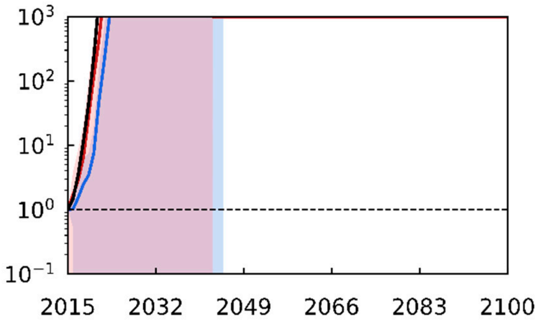	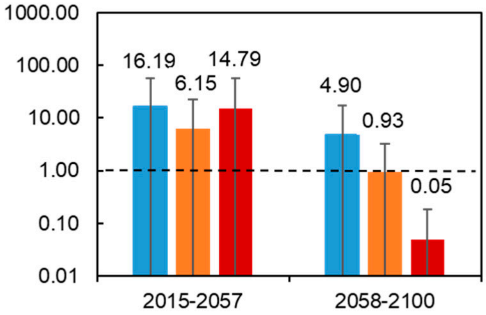
Jämtland	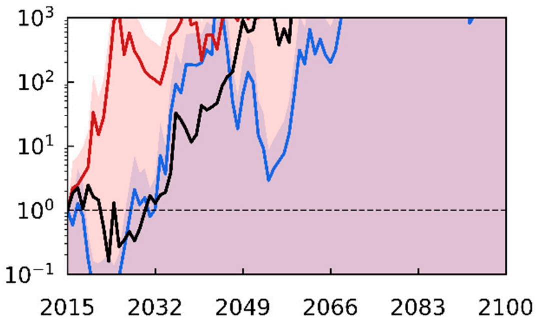	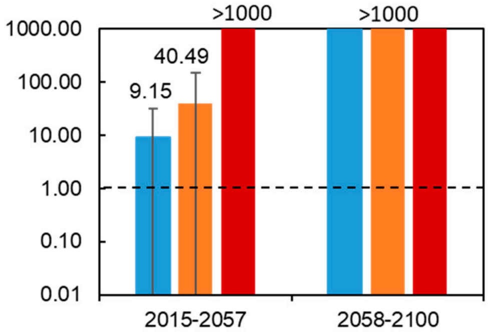
Dalarna	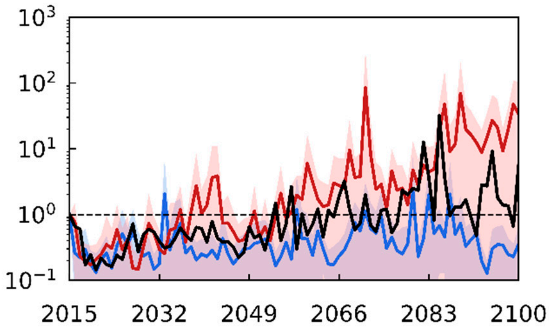	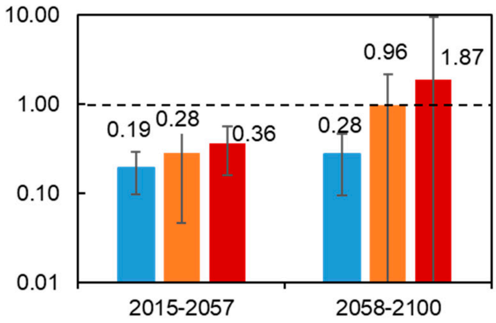
Gävleborg(Ockelbo)	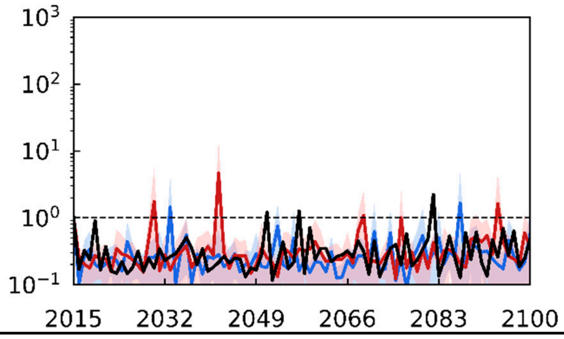	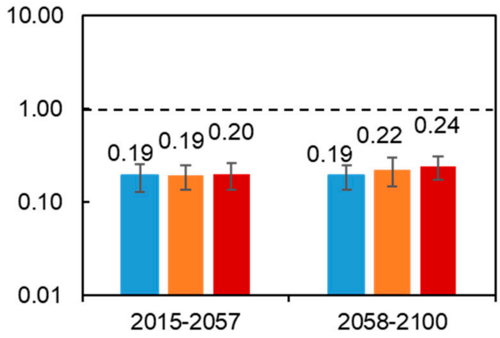

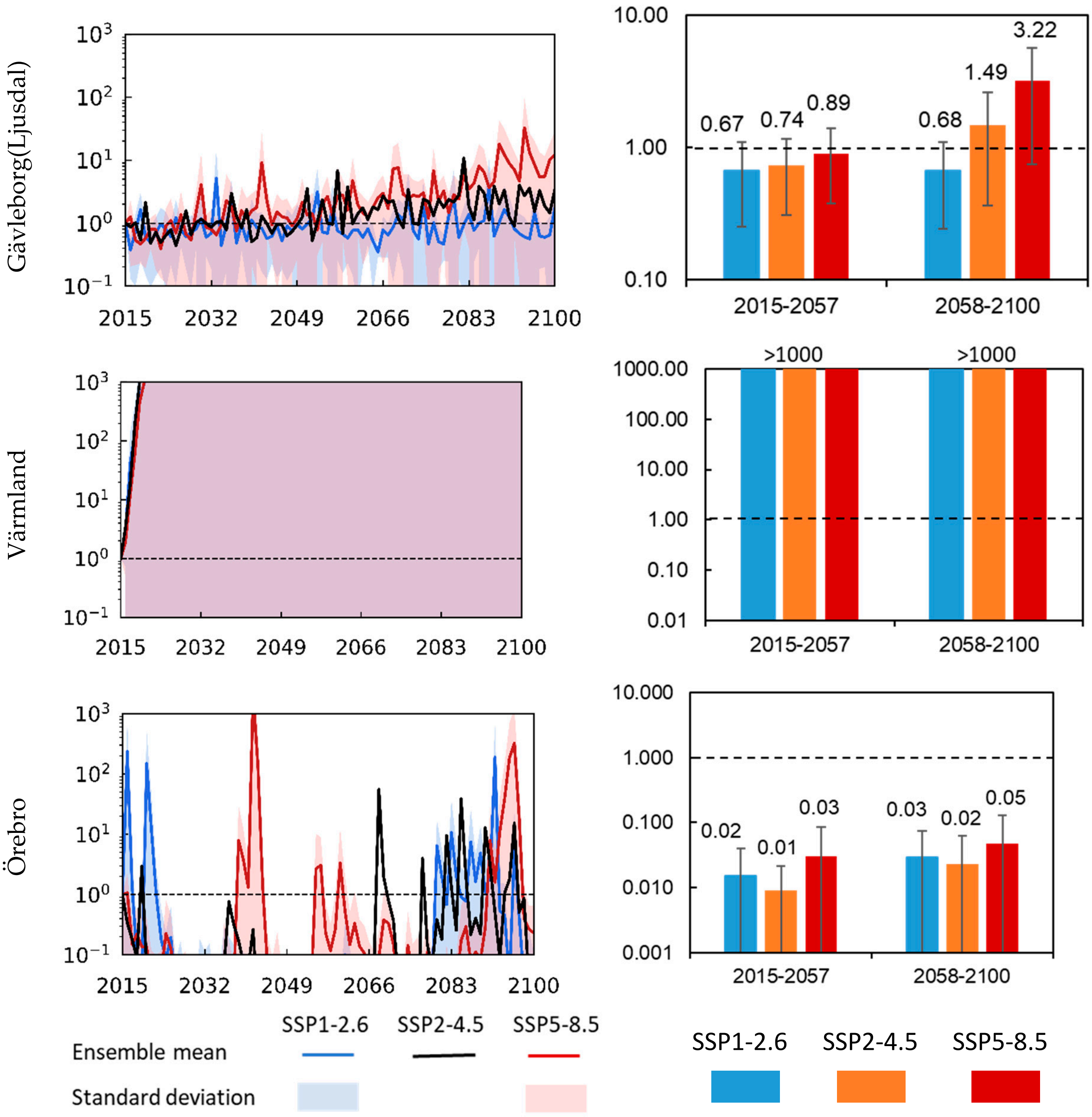

Figure 5. Normalized projected annual tularemia cases (nTul) 2015-2100 (left panel) and normalized divergence (D_L^*) or endemic levels (N_A^* or N_O^*) in the periods 2015-2057 and 2058-2100 (right panel) in Norbotten, Jämtland, Dalarna, Gävleborg (two sites), Värmland, and Örebro county in CMIP6 scenarios SSP1-2.6, SSP2-4.5, and SSP5-8.5. The solid lines (left panel) and bars (right panel) are mean values of modeled outputs. The intervals in shaded areas (left panel) and between error bars (right panel) are ranges between mean ± standared deviations.
